# Enhancement of the therapeutic efficacy of the MAP regimen using thiamine pyrophosphate‐decorated albumin nanoclusters in osteosarcoma treatment

**DOI:** 10.1002/btm2.10472

**Published:** 2022-12-26

**Authors:** So‐Yeol Yoo, Yong‐Hyeon Mun, Nae‐Won Kang, Jang Mo Koo, Dong Hwan Lee, Ji Hoon Yoo, Sang Min Lee, Seokjin Koh, Jong Chan Park, Taejung Kim, Eun Kyung Shin, Han Sol Lee, Jaehoon Sim, Keon Wook Kang, Sang Kyum Kim, Cheong‐Weon Cho, Myeong Gyu Kim, Dae‐Duk Kim, Jae‐Young Lee

**Affiliations:** ^1^ College of Pharmacy, Chungnam National University Daejeon Republic of Korea; ^2^ College of Pharmacy and Research Institute of Pharmaceutical Sciences, Seoul National University Seoul Republic of Korea; ^3^ College of Pharmacy and Graduate School of Pharmaceutical Sciences, Ewha Womans University Seoul Republic of Korea

**Keywords:** albumin nanoclusters, hydroxyapatites, MAP regimen, osteosarcoma, thiamine pyrophosphate

## Abstract

Recent studies on osteosarcoma regimens have mainly focused on modifying the combination of antineoplastic agents rather than enhancing the therapeutic efficacy of each component. Here, an albumin nanocluster (NC)‐assisted methotrexate (MTX), doxorubicin (DOX), and cisplatin (MAP) regimen with improved antitumor efficacy is presented. Human serum albumin (HSA) is decorated with thiamine pyrophosphate (TPP) to increase the affinity to the bone tumor microenvironment (TME). MTX or DOX (hydrophobic MAP components) is adsorbed to HSA‐TPP via hydrophobic interactions. MTX‐ or DOX‐adsorbed HSA‐TPP NCs exhibit 20.8‐ and 1.64‐fold higher binding affinity to hydroxyapatite, respectively, than corresponding HSA NCs, suggesting improved targeting ability to the bone TME via TPP decoration. A modified MAP regimen consisting of MTX‐ or DOX‐adsorbed HSA‐TPP NCs and free cisplatin displays a higher synergistic anticancer effect in HOS/MNNG human osteosarcoma cells than conventional MAP. TPP‐decorated NCs show 1.53‐fold higher tumor accumulation than unmodified NCs in an orthotopic osteosarcoma mouse model, indicating increased bone tumor distribution. As a result, the modified regimen more significantly suppresses tumor growth in vivo than solution‐based conventional MAP, suggesting that HSA‐TPP NC‐assisted MAP may be a promising strategy for osteosarcoma treatment.

## 1 INTRODUCTION

Current clinical guidelines for osteosarcoma management recommend combination therapy using various antineoplastic agents rather than monotherapy.^1^, ^2^ One of the preferred treatment options is the “MAP” regimen, a combination therapy of methotrexate (MTX), doxorubicin (DOX; adriamycin), and cisplatin (CDDP; *cis*‐diammine‐dichloro‐platinum (II)), the clinical availability of which has been reported.^3^, ^4^ In pursuit of more effective strategies, recent studies have mainly focused on modifying the drug combinations, including MAP + ifosfamide, MAP + ifosfamide + etoposide, and MAP + PEGylated IFN‐α‐2b.^3^, ^4^, ^5^, ^6^, ^7^ However, despite the significant progress in nanotechnology,^8^, ^9^, ^10^ there have been limited attempts to apply nano‐drug delivery systems (NDDS) to this clinical regimen, which warrants further investigation into a novel NDDS‐based MAP regimen for effective osteosarcoma treatment.

As osteosarcoma features a bone‐like mineralized extracellular matrix, NDDS with bone‐targeting ability, mostly relying on the potent binding affinity of bisphosphonate moieties to hydroxyapatite (HAp), the major mineral component of the bone, may be an effective option.^11^, ^12^, ^13^ Previously, our group reported alendronate‐decorated albumin nanoclusters (NCs) that were prepared by inducing self‐assembly of albumin molecules via ball milling‐assisted adsorption of a hydrophobic drug.^8^, ^9^, ^10^ Unlike conventional (i.e., chemically cross‐linked) albumin nanoparticles, these albumin NCs exhibit concentration‐dependent size reduction, as in the case of Abraxane (paclitaxel protein‐bound particles for injectable suspension), indicating disassembly of the NC structure upon dilution, which minimizes the off‐target accumulation and residual toxicity of NDDS.^8^, ^9^, ^10^ However, given the anti‐osteoclastic activities of bisphosphonates, bisphosphonate‐containing nanomaterials can increase the risk of osteonecrosis.^14^, ^15^ Alternatively, thiamine pyrophosphate (TPP), an endogenous derivative of vitamin B_1_, may be a safer option for the HAp‐binding moiety, as the vitamin B_1_ structure exhibits no evident toxicity even at extremely high exposure levels (e.g., 500 mg per day for 1 month) and maintains ionic interactions with HAp via the pyrophosphate group.^16^, ^17^, ^18^ Therefore, we hypothesized that surface decoration with the TPP moiety would provide NDDS with a bone tumor‐homing effect, without raising toxicity issues.

**FIGURE 1:**
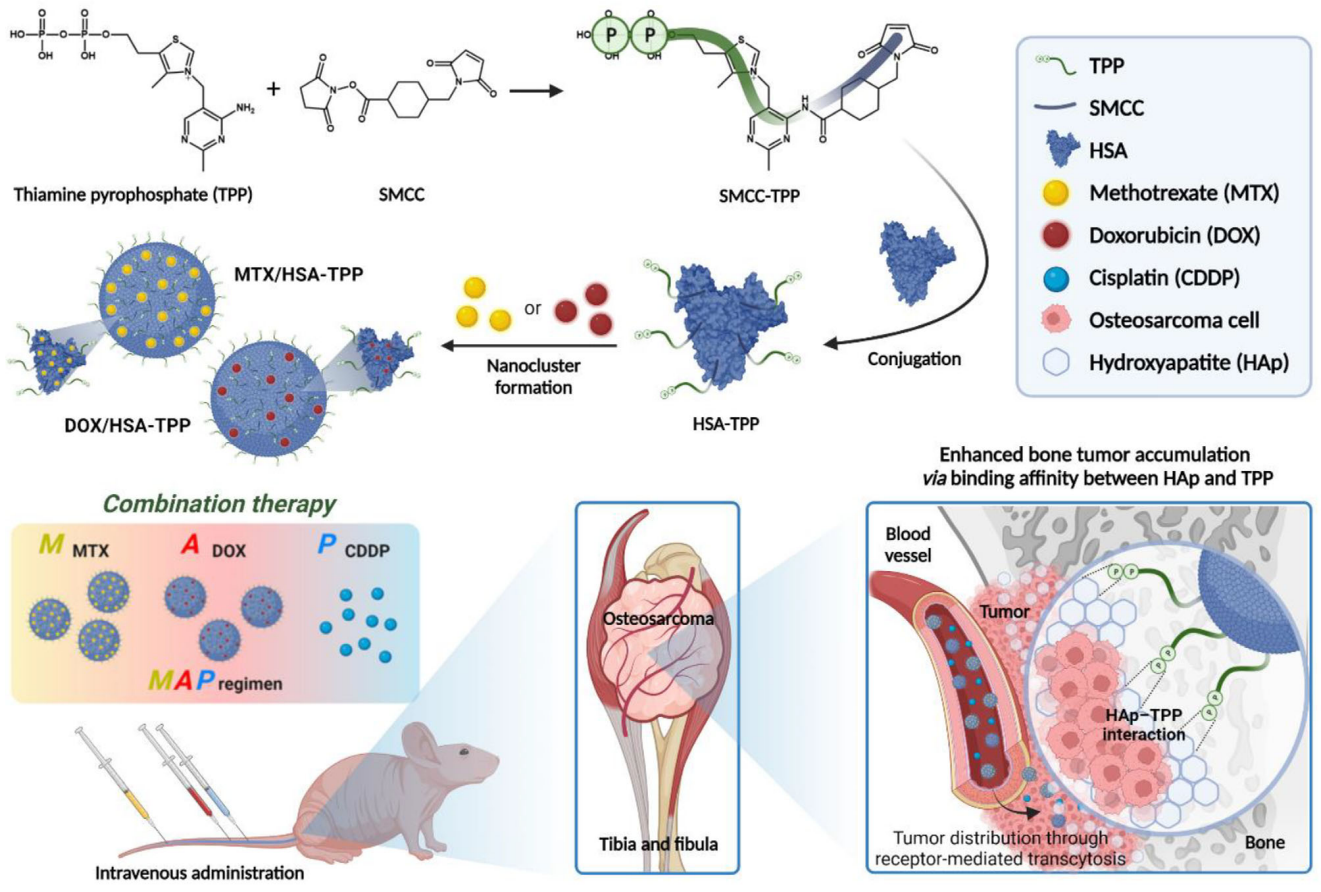
Schematic illustration of the HSA‐TPP NC‐based MAP regimen for osteosarcoma treatment (created with BioRender.com).

Previous reports on NDDS for bone tumor therapy are mostly limited to monotherapy. Here, we aimed to evaluate the therapeutic benefits of NDDS‐based combination therapy by replacing free MTX and DOX in MAP with their albumin NC surrogates. To develop albumin NCs with bone‐tumor targeting ability, human serum albumin (HSA) was adopted as a core framework because of its excellent tumor‐homing ability,^19^ and TPP was conjugated to HSA to endow targeting ability to the bone tumor microenvironment (TME) (Figure 1). In our hypothesis, TPP‐decorated HSA (HSA‐TPP) may self‐assemble into NCs upon the adsorption of DOX or MTX, the hydrophobic components of the MAP regimen, similarly to the alendronate‐modified HSA reported previously.^8^, ^9^, ^10^ The drug‐adsorbed HSA‐TPP NCs may form strong charge‐to‐charge interactions with HAp, which may translate into improved bone tumor accumulation efficiency as compared with that of unmodified HSA NCs. The improved tumor distribution would augment the synergistic effects of MTX, DOX, and CDDP, thereby enhancing the therapeutic efficacy of MAP in osteosarcoma treatment.

## 2 MATERIALS AND METHODS

### 2.1 Materials

HSA, TPP, succinimidyl‐4‐(*N*‐maleimidomethyl)cyclohexane‐1‐carboxylate (SMCC), HAp beads (5 μm, surface area ≥ 80 m^2^/g), triethylamine, and deuterium oxide (D_2_O) were purchased from Sigma‐Aldrich Co. (St. Louis, MO). Matrigel was purchased from Corning Inc. (Corning, NY). DOX hydrochloride (DOX HCl) was obtained from LC Laboratories (Woburn, MA). MTX hydrate was purchased from Tokyo Chemical Industry Co., Ltd. (Tokyo, Japan). CDDP was purchased from MedChemExpress (Monmouth Junction, NJ). Cyanine 5.5 *N*‐hydroxysuccinimide ester (Cy5.5‐NHS) was purchased from BioActs (Incheon, Republic of Korea). Eagle's minimum essential medium (MEM) and phosphate‐buffered saline (PBS) were purchased from Welgene Inc. (Gyeongsan, Republic of Korea). Fetal bovine serum (FBS), newborn calf serum (NBCS), and penicillin–streptomycin were purchased from Gibco Life Technologies, Inc. (Carlsbad, CA).

### 2.2 Synthesis and characterization of TPP‐decorated HSA

TPP was conjugated to HSA via a heterobifunctional crosslinking reaction. SMCC (2.51 mg; 7.51 μmol) dissolved in dimethyl sulfoxide (DMSO; 2 ml) was added to TPP (35 mg; 76.0 μmol) dissolved in phosphate buffer (2 ml, pH 7.0) and the mixture was stirred at 40°C for 3 h. The resulting maleimide‐activated TPP solution was added dropwise to an HSA solution (1 mg/ml; 20 ml; pH 7.6; 0.30 μmol) and the mixture was stirred at 40°C for 20 h. Then, the solution was dialyzed against double‐deionized water (DDW) using a semi‐permeable bag (molecular weight cutoff [MWCO]: 6–8 kDa; Cellu⋅Sep; Membrane Filtration Products, Inc., Seguin, TX) to remove the unconjugated TPP and linker, and the dialysis products were lyophilized at −80°C for 2 days and stored at −20°C until use.

HSA‐TPP conjugation was evaluated by sodium dodecyl sulfate‐polyacrylamide gel electrophoresis (SDS‐PAGE) using 8% gel. HSA and HSA‐TPP were electrophoresed at 80 *V* for 20 min and then at 120 *V* for 140 min. Coomassie blue staining was performed to detect the proteins. The average molecular weights of HSA and HSA‐TPP were measured by matrix‐assisted laser desorption and ionization time‐of‐flight (MALDI‐TOF) mass spectrometry (Voyager DE‐STR, Applied Biosystems, Foster City, CA), wherein the concentrations of protein samples were set at 1 mg/ml, and sinapinic acid was used as matrix. The conjugation of TPP to HSA was confirmed by phosphorus‐31 nuclear magnetic resonance (^31^P‐NMR; Avance III‐600; Bruker, Billerica, MA). The NMR samples were prepared by dissolving HSA, TPP, or HSA‐TPP in D_2_O.

### 2.3 Fabrication of drug‐adsorbed NCs

DOX base was prepared using a liquid–liquid extraction method. Briefly, DOX HCl (50 mg) dissolved in DDW (30 ml) was mixed with chloroform (20 ml) and triethylamine (25 μl) in a separatory funnel. The chloroform phase was collected and evaporated using a rotary evaporator (Rotary Evaporator N‐1300 V, EYELA, Tokyo, Japan). The precipitated DOX base was dissolved in a minimal amount of DMSO and lyophilized.

Drug‐adsorbed NCs were fabricated using an ultrasonic processor (VC‐750; Sonics & Materials, Inc., Newtown, CT). DOX base (2 mg) or MTX (3 mg) dissolved in DMSO (100 μl) and HSA or HSA‐TPP dissolved in DDW (900 μl; as protein, 83.3 μM) were vortex‐mixed gently for 30 s and dispersed by ultrasonication at an amplitude of 20% and a pulse cycle of 2 s on and 3 s off for 1 min in an ice bath. The resulting solution was lyophilized to remove the solvent, and the freeze‐dried product was resuspended in DDW (1 ml) and filtered through a syringe filter (pore size: 0.45 μm; Minisart RC15; Sartorius, Göttingen, Germany) to remove unloaded (i.e., precipitated) DOX or MTX. To prepare a CDDP solution, CDDP was dissolved in a 0.9% sodium chloride solution using ultrasonication.

### 2.4 Characterization of drug‐adsorbed NCs

The mean diameter, polydispersity index, and zeta potential (at pH 7.4) values of the drug‐adsorbed NCs were measured by dynamic light scattering (DLS) and laser Doppler electrophoresis, respectively, using a Zetasizer Ultra instrument (Malvern Instruments Ltd., Malvern, UK). The morphology of the drug‐adsorbed NCs was observed using transmission electron microscopy (TEM; JEM‐F200; JEOL, Tokyo, Japan). Resuspended NCs were placed on a 200‐mesh carbon‐coated copper grid and negatively stained with uranyl acetate. To calculate the DOX AE, DOX‐adsorbed NCs were diluted in DMSO (dilution factor: 20) and analyzed using a UV–Vis spectrophotometer (Multiskan GO; Thermo Scientific Inc., Waltham, MA) at 492 nm. The MTX adsorption efficiencies (AE) was determined using a high‐performance liquid chromatography (HPLC) system (Agilent 1260 infinity; Agilent Technologies, Palo Alto, CA) equipped with a reverse‐phase column (Kinetex C18, 4.6 × 100 mm, 2.6 μm; Phenomenex, Torrance, CA) and a guard column (C18, 4 × 2.0 mm; Phenomenex). MTX‐adsorbed NCs were diluted in a mobile phase (dilution factor: 300) consisting of potassium phosphate buffer (5 mM; pH 2.5) and acetonitrile (ACN) (85:15, *v*/*v*). The detection wavelength for MTX and flow rate were set at 303 nm and 1.0 ml/min, respectively. The injection volume and column temperature were set at 20 μl and 25°C, respectively.

### 2.5 Colloidal stability and in vitro drug release

The colloidal stability of drug‐adsorbed NCs was evaluated in a 50% FBS solution, which simulates the plasma condition. The NCs were incubated in a shaking water bath (37°C; 50 rpm), and their mean diameters were monitored at 0, 1.5, 3, 6, 9, 12, and 24 h of incubation using a Zetasizer Ultra (Malvern Instruments Ltd.).

DOX and MTX release patterns from the NCs were investigated at pH 5.5, 6.7 and 7.4. Drug‐adsorbed NCs (drug concentration: 0.5 mg/ml; 150 μl) were loaded into mini‐GeBAflex tubes (MWCO: 6–8 kDa; Gene Bio‐Application Ltd., Yavne, Israel). Each tube was immersed in release medium (2 ml; 20% NBCS; pH 5.5, 6.7, and 7.4) and incubated in a shaking water bath (50 rpm; 37°C). The NC‐filled tube was transferred to a new container filled with fresh release medium (2 ml) after 1, 3, 6, 24, and 48 h of incubation. The collected samples (50 μl) were mixed with ACN (150 μl) containing formic acid (0.1%, *v*/*v*) and an internal standard (IS; docetaxel; 100 ng/ml). The mixture was vortexed for 3 min and centrifuged at 20,378×*g* for 3 min. The supernatants were analyzed by liquid chromatography–tandem mass spectrometry. The chromatographic separation was performed using a Shimadzu Nexera XR Modular HPLC system (Shimadzu, Kyoto, Japan) equipped with a reverse‐phase column (Kinetex C18, 100 × 4.6 mm, 2.6 μm; Phenomenex) and a guard column (C18, 4 × 2.0 mm; Phenomenex) at 25°C. Elution was performed under an isocratic condition at a flow rate of 0.4 ml/min. The mobile phase was composed of ACN (0.1% formic acid, *v*/*v*) and DDW (0.2% formic acid, *v*/*v*) (70:30, *v*/*v*). The injection volume and lower limit of quantitation were 5 μl and 50 ng/ml, respectively. The ionized molecules were detected using an API 3200 system (SCIEX, Framingham, MA). The optimized parameters in the multiple reaction‐monitoring mode are summarized in Table S1. The cumulative release (*F*; %) versus time (*t*) was plotted and fitted using first‐order with *F*_max_, Hopfenberg, Korsmeyer–Peppas, and Peppas–Sahlin models, based on the following equations:

$$\text{First}-\text{order model}:F=F_{\max}∙\left(1-e^{-k∙t}\right)$$

$$\text{Hopfenberg model}:F=100∙\left[1-{\left(1-k_{\mathrm{HB}}∙t\right)}^{n}\right]$$

$$\text{Korsmeyer}–\text{Peppas model}:F=k_{\mathrm{KP}}∙t^{n}$$

$$\text{Peppas}–\text{Sahlin model}:F=k_{1}∙t^{m}+k_{2}∙t^{2m},$$

where *F*_max_ is the maximum cumulative release, and *k*, *k*_HB_, *k*_KP_, *k*_1_, and *k*_2_ are the release rate constants of the models.

### 2.6 Cell culture

In vitro cellular uptake and cytotoxicity studies were conducted in HOS/MNNG human osteosarcoma cells (CRL‐1547; ATCC, Manassas, VA). The cells were cultured in MEM containing l‐glutamine (300 mg/L) and supplemented with 10% FBS (*v*/*v*) and 1% penicillin–streptomycin (*v*/*v*) at 37°C in a 5% CO_2_ atmosphere with a relative humidity of 95%.

### 2.7 In vitro osteosarcoma accumulation studies

Tumor accumulation of the NCs was investigated in vitro using an osteosarcoma culture model consisting of HOS/MNNG cells and HAp beads. HAp beads were washed with DDW and freeze‐dried overnight before use. A HOS/MNNG monolayer culture model was prepared to investigate the interactions between HOS/MNNG cells and HAp beads. HOS/MNNG cells (5 × 10^5^/well) were seeded into 6‐well plates and cultured for 12 h, followed by a 1‐h incubation with HAp beads (2.5 mg). The cells were then washed with PBS to remove unadsorbed HAp beads and stained with alizarin red S (ARS; 50 μg/ml) for 5 min to confirm the presence of calcium. The cells were then trypsinized, and their forward scatter (FSC), side scatter (SSC), and ARS fluorescence intensity were measured by flow cytometry (NovoCyte; Agilent Technologies, Santa Clara, CA). The amount of Ca‐ARS complex formed was quantified by extracting the cells with 10% cetylpyridinium chloride solution and measuring the absorbance at 550 nm using a Multiskan GO instrument.

The scaffold‐based culture model was fabricated using a semi‐solid cell culture medium. HOS/MNNG cells (1.5 × 10^6^) and HAp beads (7.5 mg) were suspended in a mixture of cell culture medium and Matrigel (1:1, *v*/*v*). The suspension was dispersed on a confocal dish and stabilized at 37°C for 20 min to form an osteosarcoma‐mimicking gel matrix. Then, the drug‐adsorbed NCs (50 μg/ml DOX or 250 μg/ml MTX) were applied onto the gel, which was then incubated for 12 h. After the incubation, the gels were gently washed with PBS, and mounting medium composed of 0.1 M Tris/HCl buffer (pH 9.0) and glycerol (10:90, *v*/*v*) was added to the culture slides. The fluorescence intensity of DOX and MTX was detected using confocal laser scanning microscopy (LSM 880 with Airyscan; Carl‐Zeiss, Oberkochen, Germany). Total fluorescence intensity images were quantified at each color channel using the ImageJ software (Leica Camera AG, Wetzlar, Germany).

The affinity of HSA‐TPP to HAp was assessed by quantifying the amount of DOX and MTX adsorbed onto HAp by flow cytometry. DOX/HSA or DOX/HSA‐TPP (200 μg/ml DOX) was vortex‐mixed with HAp (10 mg) for 1 min. Similarly, MTX/HSA or MTX/HSA‐TPP (2 mg/ml MTX) was vortex‐mixed with HAp (0.5 mg) for 1 min. The suspensions were centrifuged at 16,000×*g* for 1 min to remove unbound NCs. The HAp pellets were resuspended in 2% FBS (*v*/*v*)‐containing PBS (pH 7.4), and DOX and MTX fluorescence intensity was measured using NovoCyte and FACSCanto II (BD Biosciences, San Jose, CA) instruments, respectively.

### 2.8 In vitro cytotoxicity studies

To measure the cytotoxicity of mono‐ and combination therapies, HOS/MNNG cells were seeded into 96‐well plates at a density of 5000 cells per well and incubated at 37°C for 24 h. For the combination therapy, the cells were treated with a drug solution or drug‐adsorbed NCs at various drug concentrations (Table S2). DOX/HSA‐TPP and CDDP solutions were added, and the plates were incubated for 24 h. Then, MTX/HSA‐TPP was added, and the plates were incubated for another 24 h. Monotherapy of each drug was conducted at different concentration ranges: 0.125, 0.25, 0.5, 1.25, 2.5, 5.0, 12.5, 25.0, and 50.0 μg/ml for DOX; 0.0005, 0.002, 0.005, 0.02, 0.05, 0.1, 1, 10, and 100 μg/ml for MTX; and 0.30, 0.75, 1.5, 3.0, 7.5, 15, 30, and 75 μg/ml for CDDP. Cell viability was evaluated using the Cell Counting Kit‐8 (Dojindo, Tokyo, Japan). The absorbance at a wavelength of 450 nm was measured using a UV–vis spectrophotometer (Multiskan GO). The half‐maximal inhibitory concentration (IC_50_) and combination index (CI) values were calculated based on dose‐effect curves and using the Chou–Talalay method, respectively.^20^

### 2.9 Animal model

Balb/c nude mice (female, 4 weeks old) were purchased from Nara Biotech (Seoul, Republic of Korea). The mice were reared in a light‐controlled room at 22 ± 2°C with a relative humidity of 40 ± 5% (Institute of Drug Research & Development, Chungnam National University, Daejeon, Republic of Korea). The animals had ad libitum access to food and water. The animal study protocol was approved by the Committee for Ethical Usage of Experimental Animals at Chungnam National University (approval number: 202103A‐CNU‐085). The orthotopic bone tumor xenograft mouse model was established by intratibial inoculation of an osteosarcoma cell suspension in the right hindleg of the mice. The suspension was prepared by suspending HOS/MNNG cells (5 × 10^6^ cells) in a mixture of culture medium and Matrigel (1:1, *v*/*v*; total volume, 100 μl). HAp beads (4 mg), washed with ethanol and dried overnight to prevent microbial contamination, were dispersed in the same medium and implanted intratumorally 10 days after HOS/MNNG inoculation to simulate the bone TME.

### 2.10 In vivo fluorescence imaging studies

The in vivo biodistribution of NCs was evaluated in a HOS/MNNG orthotopic xenograft mouse model, using fluorescence dye‐tagged NCs. Cy5.5‐NHS (100 μg) dissolved in DMSO was added to HSA (50 mg) dissolved in PBS (10 ml; pH 7.4). The mixture was stirred at room temperature for 6 h and dialyzed against DDW using a dialysis bag (MWCO: 6–8 kDa; Cellu⋅Sep). The dialysis product was concentrated and purified using a PD‐10 desalting column (GE Healthcare, Chicago, IL) to obtain Cy5.5‐labeled HSA (Cy5.5‐HSA). Cy5.5‐labeled HSA‐TPP (Cy5.5‐HSA‐TPP) was prepared by conjugating TPP to Cy5.5‐HSA according to the method described in the section “*Synthesis and Characterization of TPP‐decorated HSA*.” Cy5.5‐labeled NCs were fabricated using Cy5.5‐HSA and Cy5.5‐HSA‐TPP following the protocol described in the section “*Fabrication of Drug‐adsorbed NCs*,” of which the Cy5.5 content was analyzed by measuring the absorbance at a wavelength of 675 nm using the Multiskan GO UV–vis spectrophotometer. The tumor volume (*V*, mm^3^) was calculated from the volume difference between the tumor‐bearing leg and the normal leg using the following equations, assuming that the distal lower limbs have an ovoidal shape:

$$V_{R}=0.5\times \text{length}\times {\left(\text{width}\right)}^{2}\;\text{of the right lower}\;\mathrm{leg}\;\left(i.e.,\text{tumor}-\text{bearing}\;\mathrm{leg}\right)$$

$$V_{L}=0.5\times \text{length}\times {\left(\text{width}\right)}^{2}\;\text{of the left lower}\;\mathrm{leg}\;\left(i.e.,\text{normal}\;\mathrm{leg}\right)$$

$$\text{Tumor volume}\;\left(V,{\mathrm{mm}}^{3}\right)=V_{R}–V_{L}$$

Cy5.5‐labeled NCs were injected intravenously at a Cy5.5 dose of 35 μg/kg when the average tumor volume of the mice reached 300–350 mm^3^. Whole‐body fluorescence was monitored at 0, 1, 2, 4, 8, 12, and 24 h post injection using an VISQUE InVivo Smart imaging system (Vieworks, Anyang, Republic of Korea) equipped with the CleVue software (Vieworks) under isoflurane anesthesia. For ex vivo analyses, the mice were sacrificed 24 h after injection, and their major organs and tissues, including the liver, spleen, kidneys, heart, lungs, and both legs, were dissected and analyzed using the same instrument.

### 2.11 In vivo pharmacokinetic studies

The pharmacokinetic properties of the NCs were evaluated in Balb/c mice (female, 5 weeks old; Nara Biotech). The mice were acclimatized in a light‐controlled room at 22 ± 2°C with a relative humidity of 40 ± 5% (Institute of Drug Research & Development, Chungnam National University). The experiment protocol was approved by the Committee for Ethical Usage of Experimental Animals at Chungnam National University (approval number: 202209A‐CNU‐193). Each formulation was intravenously administered at a dose of 5 mg/kg for DOX‐adsorbed NCs or 10 mg/kg for MTX‐adsorbed NCs. Blood samples (≈120 μl) were collected from retro‐orbital sinus at 0.5, 1, 2, 4, and 8 h post‐injection in a staggered manner, followed by centrifugation at 16,000×*g* for 5 min at 4°C. Aliquots (50 μl) of the supernatant (plasma) were vortex‐mixed with ACN (150 μl) containing IS (docetaxel; 100 ng/ml) for 5 min and centrifuged at 16,000×*g* for 5 min. The amounts of DOX or MTX in the supernatants were determined according to the analytical method described in Section 2.5. The pharmacokinetic parameters were calculated using WinNonlin (version 3.1; Pharsight, Mountain View, CA), including terminal half‐life (*T*_1/2_), maximum drug concentration observed (*C*_max_), area under the plasma concentration versus time curve from time 0 to 8 h (AUC_last_), the volume of distribution at steady state (*V*_*d*_), and time‐averaged total body clearance (CL).

Tumor distribution profiles of drug‐adsorbed HSA‐TPP NCs were evaluated in the orthotopic HOS/MNNG tumor xenograft model (cf. Section 2.9). When the tumor volume reached approximately 200 mm^3^, the mice were intravenously administered with each formulation at 5 mg/kg for DOX/HSA‐TPP or 10 mg/kg for MTX/HSA‐TPP. After collecting blood samples, the mice were sacrificed at 0.5, 1, 4, and 8 h post‐injection, and tumor tissues were excised and weighed. To determine the DOX and MTX concentrations, tumor tissues were homogenized with 2‐fold the volume of DDW (33.3%, w/v). Aliquots (50 μl) of the homogenate were extracted with ACN (150 μl) containing IS (docetaxel; 100 ng/ml) for 5 min and centrifuged at 16,000×*g* for 5 min. The plasma samples at each time point were also prepared using the same method above. The amounts of DOX and MTX in the tumor tissue and plasma were quantified using the analytical method described in Section 2.5.

### 2.12 In vivo anti‐tumor efficacy

The orthotopic HOS/MNNG tumor xenograft model was established as described above. The mice were divided randomly into four groups: untreated (no intervention), drug solutions (free DOX + free MTX + free CDDP), HSA NCs (DOX/HSA + MTX/HSA + free CDDP), and HSA‐TPP NCs (DOX/HSA‐TPP + MTX/HSA‐TPP + free CDDP) groups. Tumor size and body weight were measured daily. When the tumor volume reached approximately 70 mm^3^ (day 4), the drug solutions or NCs were intravenously injected following the treatment schedule: DOX (5 mg/kg) and CDDP solution (3 mg/kg) on days 4, 8, and 13; MTX (10 mg/kg) on days 6, 10, and 15. On day 17, blood samples were collected from the retro‐orbital plexus before the mice were sacrificed. Both legs were amputated and weighed to calculate the tumor weight. Major organs, including the liver, spleen, kidneys, heart, and lungs, were excised and subjected to hematoxylin and eosin (H&E) staining. Terminal deoxynucleotidyl transferase dUTP nick‐end labeling (TUNEL) staining was performed on the leg samples, along with H&E staining. Complete blood count (CBC) tests on the collected blood samples were conducted using Scil Vet abc Plus (HORIBA, Kyoto, Japan).

### 2.13 Microarray analysis of apoptosis‐related expression in tumors

Soluble proteomes were extracted from the tumors of control (untreated) and HSA‐TPP‐treated mice. After tumor tissue lysis, total protein concentrations were determined using a BCA protein assay kit (Thermo Fisher Scientific). The expression levels of apoptosis signaling pathway‐related proteins were assessed using the Human Apoptosis Antibody Array (ab134001; Abcam, Cambridge, MA) according to the manufacturer's protocol.

### 2.14 Statistical analysis

All experiments were performed at least three times. Statistically significant differences were analyzed using the two‐tailed *t*‐test or one‐way analysis of variance (ANOVA) with Tukey's multiple comparisons test. All data are presented as mean ± SD, with significance set at *p* < 0.05. The *p* values for each experiment are provided in the figure legends.

## 3 RESULTS AND DISCUSSION

### 3.1 Synthesis and characterization of HSA‐TPP

**FIGURE 2:**
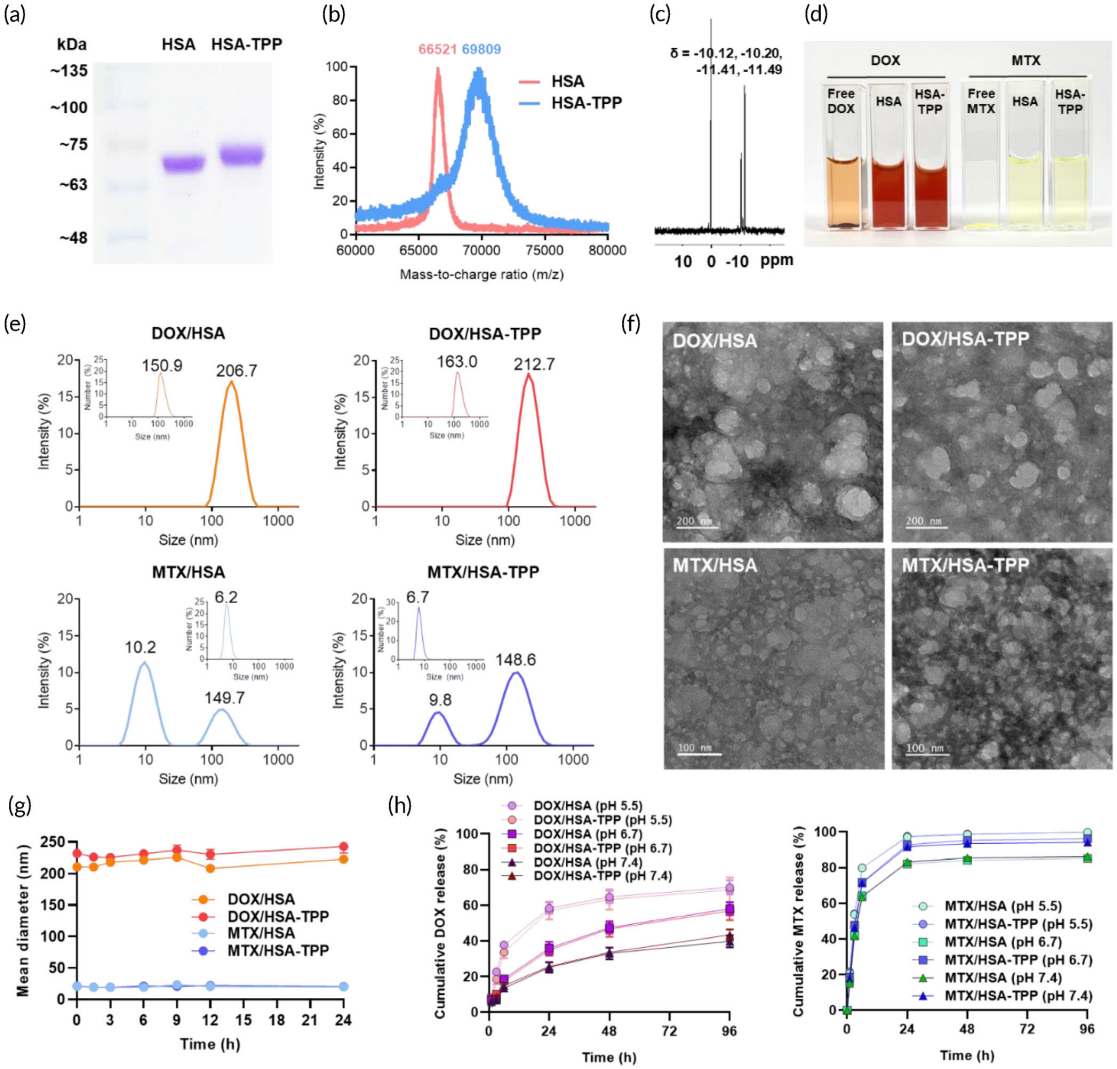
Preparation and characterization of drug‐adsorbed HSA‐TPP NCs. (a) SDS‐PAGE and (b) MALDI‐TOF analyses of native HSA and HSA‐TPP. The centroid mass‐to‐charge ratio of each peak is presented. (c) ^31^P‐NMR spectrum of HSA‐TPP to verify the introduction of the pyrophosphate group. (d) Visual appearance of aqueous suspensions of free drug and drug‐adsorbed NCs at a drug concentration of 50 μg/ml. Free DOX and MTX showed precipitation. (e) Intensity‐weighted and number‐weighted (inset) size distribution diagrams. The MND value of each peak is presented. (f) TEM observation of negatively stained NC samples. The scale bars are 200 nm for DOX‐adsorbed NCs and 100 nm for MTX‐adsorbed NCs. (g) Colloidal stability of NCs in 50% FBS for 24 h. (h) Drug release profiles of DOX‐ or MTX‐adsorbed NCs in 20% NBCS at pH 5.5, 6.7, and 7.4 for 96 h.

HSA‐TPP was synthesized using SMCC, a heterobifunctional crosslinker with both succinimide and maleimide groups. First, TPP was reacted with SMCC at pH 7.0 to induce the nucleophilic substitution of succinimide with the amine group of TPP, leaving the maleimide moiety. Subsequently, the maleimide‐activated TPP was conjugated to HSA, maintaining a pH above 7.5 so as to allow the maleimide to react with both the free thiol and amine groups of HSA.^21^ An increase in the molecular weight of HSA after TPP conjugation was confirmed via SDS‐PAGE and MALDI‐TOF analyses. Coomassie blue staining of the electrophoresed gel sample revealed that the HSA‐TPP band was shifted upward and broadened as compared with that of HSA (Figure 2a). Accordingly, in the MALDI‐TOF spectra, HSA‐TPP exhibited a higher centroid mass‐to‐charge ratio (*m/z* 69,507 ± 434) and a broader peak than native HSA (*m/z* 66,521) (Figure 2b). Considering the molecular weight of maleimide‐activated TPP (644.55 g/mol), the average number of conjugated TPP molecules per HSA molecule was calculated to be 4.63 ± 0.67.

^31^P‐NMR analysis confirmed the introduction of the TPP moiety to HSA, where HSA‐TPP exhibited a doublet of doublets signal characteristic of pyrophosphate at −10.12, −10.20, −11.41, and −11.49 ppm (Figure 2c), whereas native HSA showed no significant phosphorus signal (Figure S1). The ^31^P‐NMR spectrum of free TPP showed pyrophosphate peaks at −11.10, −11.18, −11.68, and −11.77 ppm (Figure S1). In the HSA‐TPP spectrum, the singlet at 0.20 ppm (orthophosphate) can be attributed to the phosphate salt remaining after the synthesis (i.e., phosphate buffer as a solvent).

### 3.2 Preparation and characterization of drug‐adsorbed HSA‐TPP NCs

Among the components of the MAP regimen, DOX and MTX were selected as cargo molecules of the albumin derivatives because of their hydrophobicity. Briefly, a homogeneous solid dispersion of the drug (DOX or MTX) and albumin (HSA or HSA‐TPP) was prepared via lyophilization, followed by the generation of a drug‐adsorbed albumin suspension by dispersing the lyophilizate in an aqueous medium via sonication. Drug adsorption to the albumin scaffold significantly increased the apparent solubility (Figure 2d), indicating that the protein binding mechanism can serve as an efficient drug loading method. However, the drug AE (i.e., encapsulation efficiencies) of DOX‐ and MTX‐adsorbed NCs were significantly different (Table 1). DOX/HSA and DOX/HSA‐TPP exhibited AE values of 33.4 ± 0.6% and 47.4 ± 7.1%, respectively, implying that significant amounts of large NCs may form after DOX adsorption and be removed during the filtration procedure. However, MTX/HSA and MTX/HSA‐TPP showed AE values of 80.1 ± 0.3% and 95.5 ± 1.0%, respectively, which implies that most of the NCs generated after MTX adsorption possessed injectable particle sizes (<0.45 μm).

**TABLE 1:** Physicochemical properties of drug‐adsorbed HSA‐TPP NCs.

Composition	Mean diameter (nm)	Polydispersity index	Zeta potential (mV)	Drug adsorption efficiency (%)^a^	Drug content (%)^b^
DOX/HSA	188.7 ± 6.6	0.11 ± 0.05	−22.5 ± 0.7	33.4 ± 0.6	9.5 ± 0.2
DOX/HSA‐TPP	195.0 ± 6.6	0.07 ± 0.02	−30.0 ± 0.9	47.4 ± 7.1	13.1 ± 2.0
MTX/HSA	14.2 ± 0.6	0.59 ± 0.08	−12.0 ± 1.5	80.1 ± 0.3	30.0 ± 0.1
MTX/HSA‐TPP	39.1 ± 0.3	0.85 ± 0.08	−23.3 ± 3.8	95.5 ± 1.0	34.7 ± 0.3

*Note*: Data are presented as mean ± SD (*n* = 3).

^a^ Drug adsorption efficiency (%) = (actual amount of drug in NCs) × 100/(theoretical amount of drug in NCs).

^b^ Drug content (%) = (amount of drug in NCs) × 100/(total amount of NCs).

This tendency was in line with particle size data obtained using DLS and TEM. The adsorption of the hydrophobic drugs induced nanoscale clustering of the albumin molecules. Figure 2e shows intensity‐ and number‐weighted size distributions of the prepared albumin NCs. Both DOX/HSA and DOX/HSA‐TPP exhibited a unimodal size distribution. The mean intensity‐weighted diameters (MIDs) and corresponding polydispersity index values are listed in Table 1. MTX/HSA and MTX/HSA‐TPP had MIDs of around 15 and 40 nm and displayed bimodality in the intensity‐weighted size distribution diagrams (Figure 2e). Similarly to Abraxane® and our previous formulation,^8^, ^9^, ^10^ the peaks corresponding to larger particle sizes (>100 nm) suggest the clustering of the drug‐adsorbed albumin derivative, whereas the peaks at around 10 nm imply the presence of a molecularly dispersed portion (cf. MID of native HSA ≈ 6 nm). Interestingly, the mean number‐weighted diameter (MND) of DOX‐adsorbed NCs exceeded 100 nm, whereas the MNDs of MTX‐adsorbed NCs were mostly below 10 nm (inset of Figure 2e). The difference was further investigated by TEM (Figure 2f). Large aggregates (>100 nm) were observed in DOX‐adsorbed NCs, whereas substantially smaller particles (<20 nm) were dominant in MTX‐adsorbed NCs, which is in good accordance with the respective number‐weighted size distribution plots.

The colloidal stability of each formulation was evaluated by monitoring time‐course changes in MID in 50% FBS (Figure 2g). All groups maintained their initial MID without showing any sign of aggregation or precipitation over 24 h, suggesting negligible non‐specific adsorption of serum components or self‐aggregation after intravenous injection. The zeta potentials of DOX/HSA and MTX/HSA at physiological pH exhibited negative values of −22.5 ± 0.7 and −12.0 ± 1.5 mV, respectively (Table 1). The NCs became more negatively charged when decorated with TPP, reaching −30.0 ± 0.9 and −23.3 ± 3.8 mV for DOX/HSA‐TPP and MTX/HSA‐TPP, respectively. The colloidal stability in serum can be attributed to the negative surface charge of the NCs.

Drug release from the NCs was evaluated in NBCS adjusted to pH 5.5, 6.7, and 7.4, representing the endolysosomal, acidic TME, and plasma conditions, respectively (Figure 2h).^22^, ^23^ DOX‐adsorbed NCs exhibited sustained and pH‐dependent drug release patterns. The cumulative drug release of DOX/HSA‐TPP at 96 h was calculated to be 43.5 ± 3.1% at pH 7.4, 56.8 ± 5.1% at pH 6.7, and 68.7 ± 6.9% at pH 5.5. This release pattern suggests that the DOX exposure would be more significant in the bone TME than in normal tissues. Similarly, MTX‐adsorbed NCs showed sustained drug release patterns for 96 h; however, their release was faster than that of DOX‐adsorbed NCs. To further investigate the release pattern, each release profile was fitted to four different release kinetics models (Table S3). The DOX release profiles showed the highest correlation coefficients (*R* ^2^) when fitted to the Peppas–Sahlin model, indicating that drug release may follow a mixed pattern of Fickian diffusion and case II relaxation, representing DOX diffusion from NCs and concomitant disassembly of the NC structure by the removal of DOX, respectively.^24^, ^25^ However, the best fit of the MTX release profiles was observed with the first‐order model, implying that MTX release may follow a simple drug‐protein dissociation model.^26^ These results support that DOX adsorption may induce stronger clustering of albumin molecules than MTX adsorption, as corroborated by the DLS and TEM analyses (Figure 2e, f).

### 3.3 Affinity of HSA‐TPP NCs to the bone TME

**FIGURE 3:**
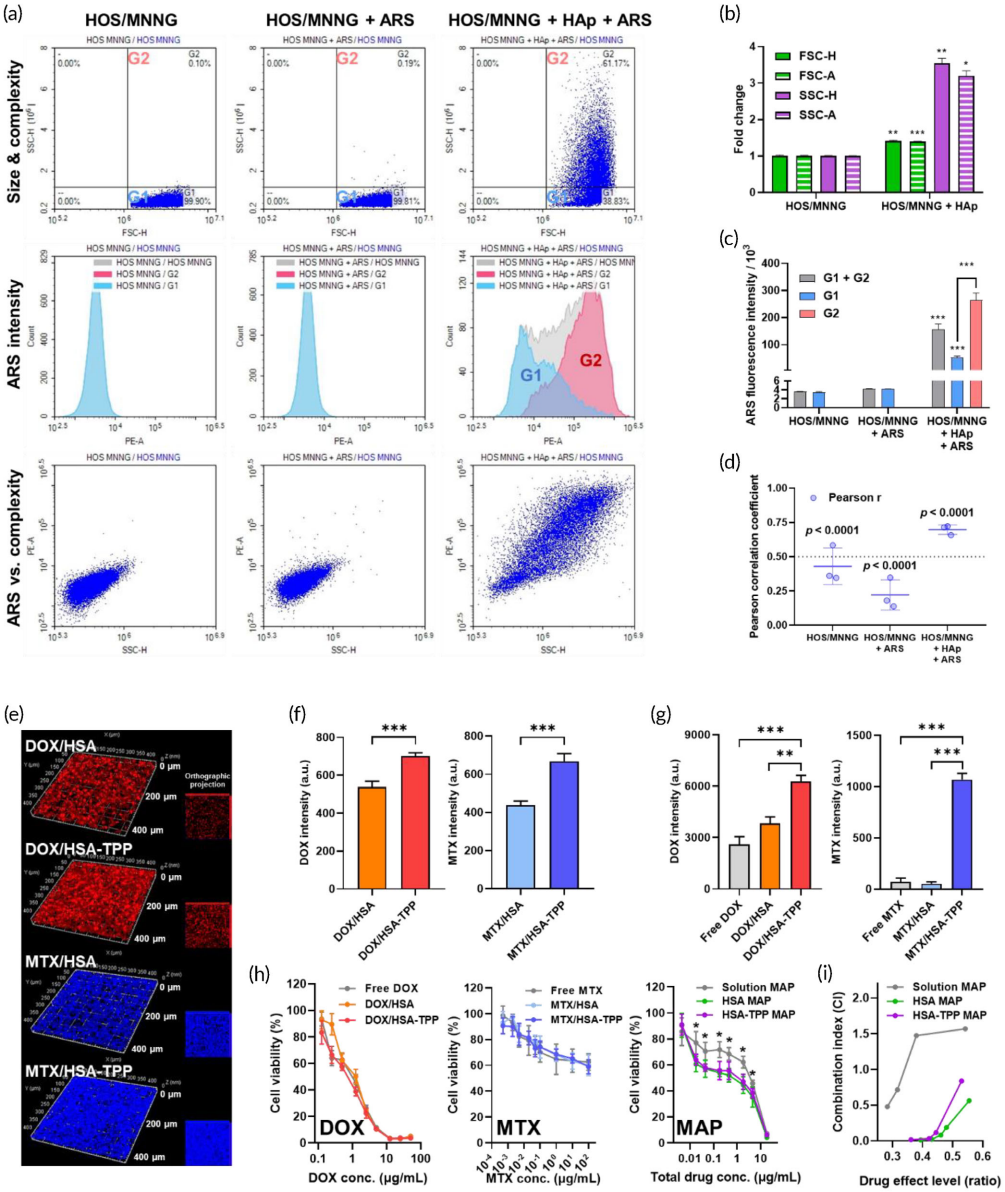
Affinity to the bone TME and in vitro antitumor efficacy of the NC‐assisted MAP regimen. (a) Flow cytometry analysis of HOS/MNNG cells cultured in the presence of HAp. ARS was used as a calcium staining dye. (b) Fold‐changes in FSC and SSC of HOS/MNNG cells after incubation with HAp beads. (c) Mean ARS fluorescence intensity of SSC‐low (G1) and SSC‐high (G2) subsets. (d) Pearson correlation coefficient between ARS fluorescence intensity and SSC‐H value. (e) Z‐stacked confocal microscopic images of a scaffold‐cultured osteosarcoma model after a 12‐h incubation with the developed NCs. Red and blue represent the fluorescence of DOX and MTX, respectively. Corresponding orthographic projection images are presented on the right side, based on which area‐averaged total fluorescence intensity (f) was quantified by image analysis. (g) Flow cytometry analysis of HAp beads after incubation with free drug or drug‐adsorbed NCs. (h) In vitro antitumor efficacy of monotherapy and MAP against HOS/MNNG cells, based on which CI values at observed drug effect levels (i) were calculated. *p < 0.01, **p < 0.001, and ***p < 0.0001.

To investigate the affinity of the NCs to the bone TME in vitro, we attempted to establish an osteosarcoma culture model using HAp beads. Physical interactions between human osteosarcoma cells (HOS/MNNG) and bone mineral (HAp) were preliminarily investigated in a monolayer culture model using flow cytometry. Singlet gates of HOS/MNNG and HAp were determined based on forward scatter area (FSC‐A) and height (FSC‐H), by which the cells and HAp beads were efficiently distinguished (Figure S2a). Then, the size and complexity of HOS/MNNG cells in the presence and absence of HAp were evaluated by monitoring FSC and SSC signals, respectively (Figure 3a). Interestingly, HOS/MNNG cells cultured in the presence of HAp exhibited 1.42‐ and 3.55‐fold higher FSC‐H and SSC‐H, respectively, than HOS/MNNG cells cultured in the absence of HAp, implying possible physical interactions of the cells with the HAp beads, such as cell surface adsorption (Figure 3b).

To confirm whether these changes resulted from HAp adsorption, calcium staining using ARS was performed, and the fluorescence intensity of the Ca‐ARS complex formed on cells was quantified by flow cytometry. As shown in Figure 3c, the total ARS intensity of HOS/MNNG cells cultured in the presence of HAp was 36.9‐fold higher, whereas that of HOS/MNNG cells cultured in the absence of HAp was nearly the same (1.19‐fold) as that of blank cell samples (i.e., without ARS treatment). Notably, the high‐SSC subset (G2) contributed more to the increase in ARS intensity than the low‐SSC subset (G1) (Figure 3a, c). Therefore, we performed a correlation analysis between ARS intensity and SSC‐H. The Pearson correlation coefficient of the HOS/MNNG + HAp + ARS group was 0.70 ± 0.04 (*p* < 0.0001), suggesting a high correlation (Figure 3d). The adsorbed ARS was extracted using cetylpyridinium chloride solution and quantified, which revealed that 57.7 ± 3.8% of the added HAp beads interacted with the cells (Figure S2b). Together, these results indicated that HOS/MNNG cells cultured in the presence of HAp can be exploited as an osteosarcoma model, simulating the calcification of the bone TME.

Encouraged by the above results, we developed a scaffold‐based culture model by culturing HOS/MNNG cells in the presence of HAp beads in a Matrigel®‐based semi‐solid cell culture medium. The model was used to evaluate NC affinity to the bone TME and cellular uptake profiles simultaneously. After incubation with the NCs for 12 h, the DOX and MTX fluorescence intensities of the model were measured at different focal planes (Z‐stacked) using a confocal microscope (Figure 3e). The DOX/HSA‐TPP and MTX/HSA‐TPP groups exhibited a significantly higher total fluorescence intensity than DOX/HSA (1.85‐fold) and MTX/HSA (1.49‐fold) groups, respectively (Figure 3f). In a conventional monolayer culture model without HAp, HSA‐TPP NCs exhibited a significantly higher cellular uptake efficiency in a relatively short incubation time (~1 h) than HSA NCs (1.50‐fold) (Figure S2c). This result may be partly due to albumin‐receptor‐mediated endocytosis, considering the comparable uptake efficiencies of HSA and HSA‐TPP NCs in the presence of free HSA as a competitive cellular uptake inhibitor.^8^, ^9^, ^10^ However, under a longer incubation time (~4 h), the difference in cellular uptake efficiency between the HSA and HSA‐TPP NCs became negligible. Therefore, the improved DOX and MTX disposition by HSA‐TPP NCs after 12 h of incubation in the osteosarcoma model (Figure 3e) can be explained by TPP–HAp interaction.

To confirm the binding affinity of TPP‐decorated NCs to the bone mineral, HAp beads were directly incubated with the NCs and analyzed by flow cytometry (Figure 3g). HAp beads incubated with DOX/HSA‐TPP showed 1.64‐ and 2.41‐fold higher DOX intensity than those incubated with DOX/HSA and free DOX, respectively. Notably, HAp beads incubated with MTX/HSA‐TPP exhibited a significantly higher MTX intensity than those incubated with MTX/HSA (20.8‐fold) and free MTX (14.9‐fold). The increased drug adsorption to HAp may result from increased ionic interactions between the TPP moiety and HAp, suggesting an enhanced targeting ability to the bone TME.

### 3.4 In vitro antitumor efficacy of HSA‐TPP NC‐assisted MAP

The in vitro antitumor efficacy of HSA‐TPP NC‐assisted MAP was compared with that of conventional solution‐based MAP in HOS/MNNG cells using a cell proliferation assay (Figure 3h). Under monotherapy conditions, DOX solution, DOX/HSA, and DOX/HSA‐TPP exhibited similar IC_50_ values of 0.829 ± 0.076 μg/ml, 0.970 ± 0.090 μg/ml, and 0.679 ± 0.082 μg/ml, respectively. MTX solution and MTX‐adsorbed NCs displayed comparable cytotoxicity over a wide range of MTX concentrations from 0.5 ng/ml to 100 μg/ml. The IC_50_ value of CDDP solution was 1.825 ± 0.444 μg/ml (Figure S3). In combination therapy, both DOX (solution or NCs) and CDDP (solution) were administered first for 24 h, and MTX (solution or NCs) was then added for another 24 h, according to the general dosing sequence of the MAP regimen.^27^ The concentration ratio of DOX/MTX/CDDP was fixed at 5/10/3, following the general dosing ratio of MAP (Table S2). Interestingly, both HSA NCs and HSA‐TPP NCs significantly lowered cancer cell viability over a total drug concentration range of 0.018–4.5 μg/ml, exhibiting a characteristic double‐sigmoidal curve shape, with IC_50_ values based on the total drug concentration being 0.17‐ and 0.37‐fold that in the solution group, respectively (Table S4).

Based on these results, CI values were calculated using the Chou–Talalay method to quantify the synergistic effects of the regimens (Figure 3i). The predicted CI values at the 50% drug effect level (CI_50_) are summarized in Table S4. The solution group showed a CI of 1.570, indicating no observable synergism between the MAP entities.^28^, ^29^ However, the HSA NC and HSA‐TPP NC groups exhibited synergistic anticancer effects, with CI_50_ values of 0.358 and 0.806, respectively. The drug‐synergizing property of albumin‐based nanoparticles has been previously reported,^30^ supporting the improved therapeutic effect of the albumin NC‐assisted MAP regimen.

### 3.5 In vivo biodistribution study in an orthotopic osteosarcoma model

**FIGURE 4:**
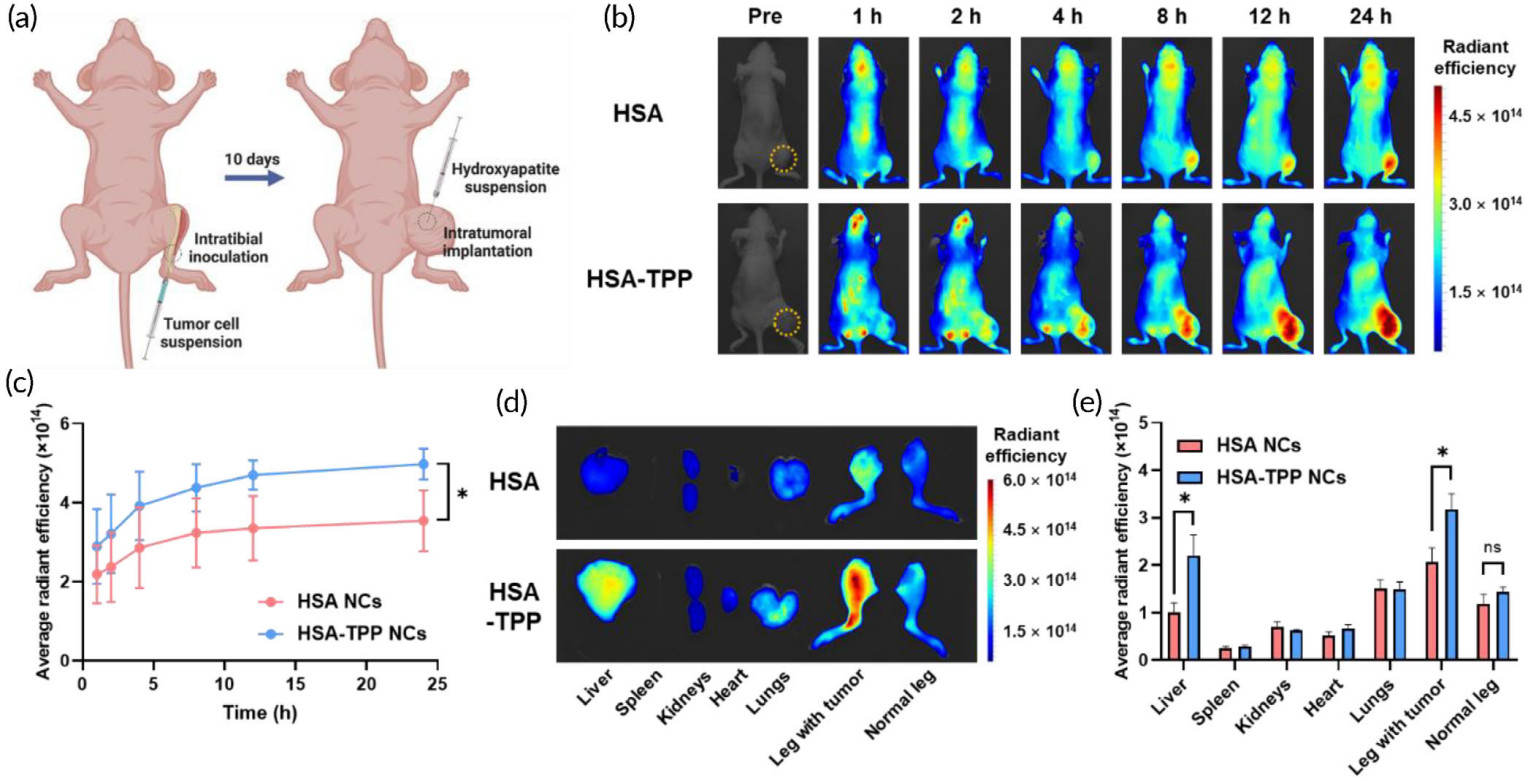
In vivo biodistribution study by NIRF imaging (*n* = 3). (a) Scheme of orthotopic osteosarcoma mouse model establishment (created with BioRender.com). (b) Time‐course whole‐body scanning images after intravenous injection of Cy5.5‐labeled NCs. Dotted circles indicate tumor regions. (c) Average RE of the tumor region in function of elapsed time post injection. (d) Representative ex vivo NIRF images of major organs and legs at 24 h post injection (images of all subjects are presented in Figure S4), and (e) corresponding average RE values. *p < 0.05; ns, not significant.

An orthotopic osteosarcoma mouse model was established to evaluate the in vivo biodistribution of HSA‐TPP NCs. HOS/MNNG cells were inoculated into the right leg via intratibial injection, followed by intratumoral implantation of HAp beads after tumor growth for 10 days, simulating the calcified extracellular matrix of the bone TME (Figure 4a). According to the calcium phosphate content of osteoblastic osteosarcoma tissue (2.4–3.9%),^11^, ^12^, ^13^ the amount of implanted HAp beads was set to 4 mg per tumor. At that concentration, the viability of HOS/MNNG cells was unaffected; HOS/MNNG cells exhibited 103.7 ± 0.6% viability at a HAp concentration of 50 μg per 10^4^ cells, which is 6.25‐fold higher than the maximum concentration in vivo (i.e., 4000 μg per 5 × 10^6^ cells).

Cy5.5‐labeled NCs were intravenously injected into the mice, and whole‐body scanning was performed at predetermined time points using near‐infrared fluorescence (NIRF) imaging (Figure 4b). HSA NCs gradually accumulated in osteosarcoma over 24 h, which can be explained by the intrinsic tumor‐homing property of albumin‐based nanocarriers.^19^ Notably, decoration with TPP increased the tumor accumulation at 24 h by 1.40‐fold (Figure 4c). Ex vivo imaging of tumor in the legs and major organs confirmed the enhanced tumor distribution of the TPP‐decorated NCs (Figures 4d and S4), where the radiant efficiency (RE) of tumor‐bearing legs in the HSA‐TPP NC‐treated group was 1.53‐fold higher than that in the HSA NC‐treated group (Figure 4e). Although the HSA‐TPP NC‐treated group showed a slightly increased mean RE in normal legs, resulting from the stronger signals in the bone and cartilage regions than those in the HSA NC‐treated group, no statistically significant difference was found between the two groups (Figure 4D). However, HSA‐TPP NCs exhibited significantly increased accumulation in the liver compared with that of HSA NCs, which could be explained by the augmented affinity to gp18 and gp30, which are expressed ubiquitously in the body, after surface modification of albumin.^31^ In addition to the NIRF imaging, we found no significant difference in blood circulation half‐life between HSA and HSA‐TPP NCs in the pharmacokinetic study performed in mice (Figure S5 and Table S5), suggesting that the enhanced tumor distribution of HSA‐TPP NCs can be attributed to the targeting ability of the decorated TPP moiety.

### 3.6 HSA‐TPP NC‐assisted MAP in an orthotopic osteosarcoma model

**FIGURE 5:**
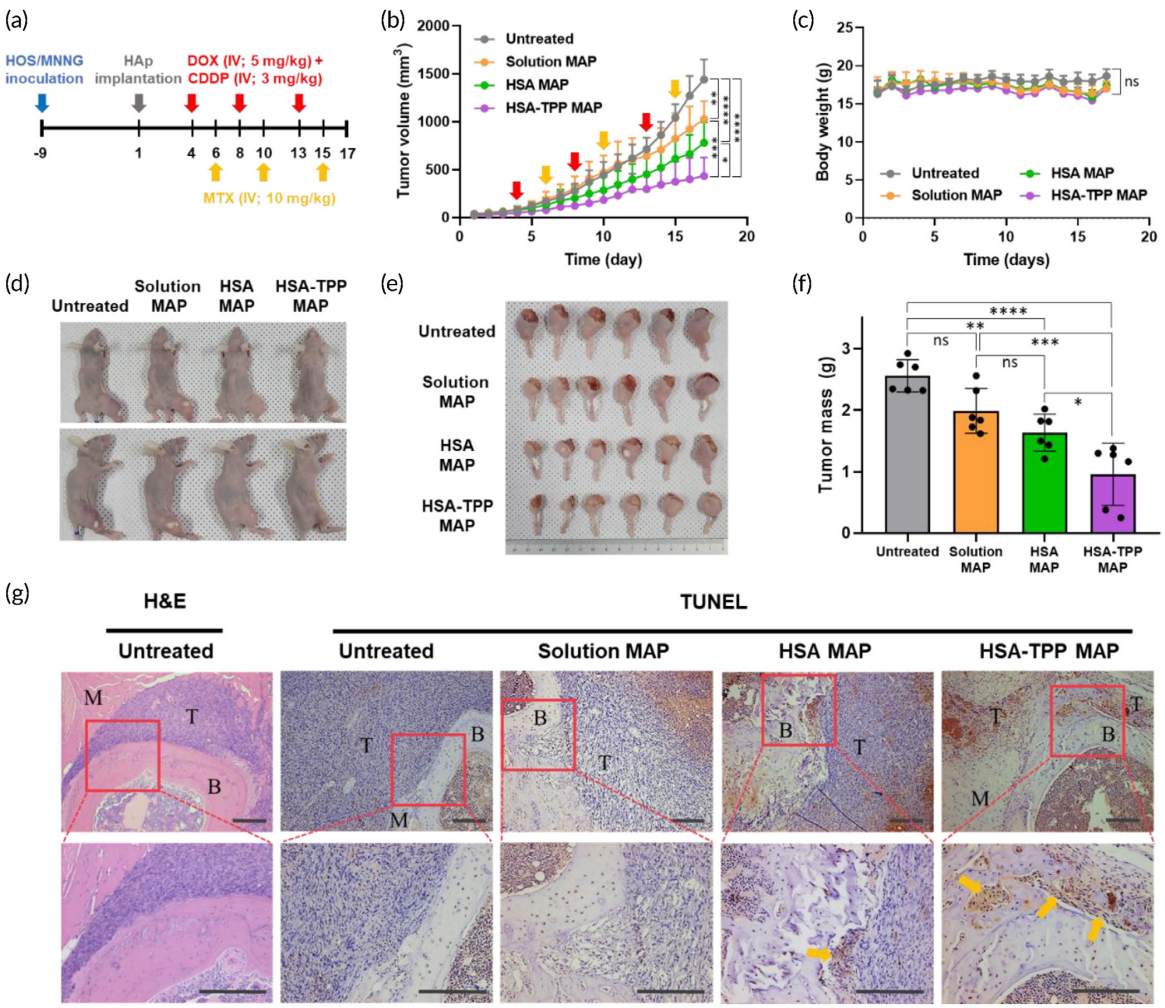
In vivo antitumor efficacy study in the orthotopic osteosarcoma mouse model. (a) Schedule of model establishment and MAP therapy. (b) Tumor growth profiles over 17 days. Tumor volume (mm^3^) was measured daily. (c) Body weight profiles of the mice over the treatment schedule. Digital images of representative mice of each group (d) and excised tumor‐bearing legs (e) on day 17. (f) Tumor mass in each group was calculated by subtracting the weight of the normal leg from that of the tumor‐bearing leg. (g) Histological analysis of the tumor‐bearing legs. B, bone; M, muscle; T, tumor. The scale bar is 100 μm.

The tumor‐suppressive effect of HSA‐TPP NC‐assisted MAP was compared with that of conventional free drug‐based MAP in the orthotopic osteosarcoma‐xenografted mouse model. Tumor‐bearing mice were randomly divided into four groups: untreated (no intervention), solution MAP (all drugs administered as free drug solutions), HSA MAP (DOX/HSA, MTX/HSA, and free CDDP), and HSA‐TPP MAP (DOX/HSA‐TPP, MTX/HSA‐TPP, and free CDDP) groups, and each intervention was administered as scheduled in Figure 5a. As DOX/HSA‐TPP exhibited a significantly longer tumor retention profile than MTX/HSA‐TPP (Figure S6), the DOX administration schedule was set to precede MTX treatment, expecting simultaneous exposure of both drugs to the tumor tissues. After three cycles of MAP, tumor growth was moderately suppressed in the solution MAP group, with a 1.40‐fold lower volume than that in the untreated group on day 17 (Figure 5b). HSA MAP further reduced the tumor volume by 1.84‐fold, which can be attributed to the innate tumor‐targeting ability of albumin. Notably, HSA‐TPP MAP substantially suppressed tumor growth, with a significantly lower tumor volume on day 17 (2.37‐ and 1.80‐fold decreases compared with the solution MAP and HSA MAP groups, respectively). No significant body weight changes were observed throughout the experiment, suggesting negligible systemic toxicities (Figure 5c). On day 17, the mice were sacrificed, and both legs of the mice in each group were excised (Figure 5d, e). The tendency of tumor weight data corresponded well with tumor volume data and confirmed that HSA‐TPP MAP had the highest tumor‐suppressive effect among the interventions (Figure 5f).

To investigate the degree of apoptosis in the tumor tissues, tumor‐bearing legs excised on day 17 were stained by TUNEL to visualize the apoptotic region^32^ and with H&E for clear morphological differentiation of the muscle, bone, and tumor tissues (Figure 5g).^33^ In the tumors of the untreated group, no observable apoptotic region was found. Although the solution MAP and HSA MAP groups exhibited apoptotic regions (brown color), these areas were mostly located far from the bone region, which can be a target site in the clinical situation. Of note, the HSA‐TPP MAP group showed significantly larger apoptotic areas, especially near the bone–tumor boundaries, which can be attributed to the increased affinity of the NCs to the bone minerals owing to the TPP decoration. The reduced expression of anti‐apoptotic proteins in tumors of the HSA‐TPP MAP group corroborated the increased apoptosis in the tumor tissues (Figure S7).

### 3.7 Toxicity profiles of HSA‐TPP NC‐assisted MAP

Toxicity profiles of HSA‐TPP NC‐assisted MAP were assessed in the orthotopic osteosarcoma‐xenografted mice. To evaluate off‐target organ toxicity, the major organs were excised and stained with H&E to investigate histological changes (Figure S8). In the HSA MAP and HSA‐TPP MAP groups, the cardiac muscle tissue, which is known to be susceptible to DOX,^34^, ^35^ displayed no observable pathological changes after three cycles of MAP when compared with the untreated group. However, mice in the solution MAP group exhibited a reduction in myofibril density with loss of striations, indicating severe cardiotoxicity. The decreased DOX‐related cardiotoxicity in the NC‐assisted MAP groups can be explained by the relatively low NC distribution to the heart (Figure 4e). Toxicities in other organs, including the liver, lungs, spleen, and kidneys, were negligible in all groups.

The enhanced safety of NC‐based MAP was confirmed by systemic toxicity evaluation. Blood samples were collected after three cycles of MAP, and CBC and serum biomarkers were assessed (Table 2).^36^ The HSA‐TPP NC‐assisted MAP groups exhibited no clinically significant changes in any of the biomarkers as compared with reference values, indicating negligible toxicities, including bone marrow suppression, which can be caused by bone‐targeted delivery of anticancer agents.^37^

**TABLE 2:** CBC and serum biochemistry parameters after three cycles of MAP

Parameters (unit)	Untreated	Solution MAP	HSA MAP	HSA‐TPP MAP
Complete blood counts				
WBC (10^3^/μl)	6.4 ± 2.1	4.9 ± 2.8	6.7 ± 4.2	5.8 ± 2.8
RBC (10^6^/μl)	10.5 ± 1.0	11.0 ± 3.1	10.2 ± 3.1	11.3 ± 2.5
HGB (g/dl)	16.5 ± 1.2	17.9 ± 4.7	14.6 ± 3.3	17.3 ± 3.9
HCT (%)	51.7 ± 3.6	54.1 ± 15.4	46.1 ± 11.8	55.7 ± 13.4
MCV (μm^3^)	48.3 ± 1.2	49.5 ± 0.5	49.0 ± 1.4	49.0 ± 1.5
MCH (pg)	15.5 ± 0.8	15.5 ± 0.3	15.7 ± 0.6	15.3 ± 0.5
MCHC (g/dl)	32.0 ± 1.0	31.6 ± 0.8	32.0 ± 1.8	31.2 ± 0.7
PLT (10^3^/μl)	129 ± 59	133 ± 75	118 ± 59	168 ± 83
Serum biochemistry				
AST (U/L)	107.3 ± 19.4	106.7 ± 26.0	107.7 ± 20.8	106.3 ± 19.2
ALT (U/L)	17.5 ± 6.4	20.5 ± 4.4	17.7 ± 1.5	18.3 ± 5.2
SCr (mg/dl)	0.48 ± 0.16	0.26 ± 0.11	0.17 ± 0.07	0.21 ± 0.09
BUN (mg/dl)	29.6 ± 6.7	38.5 ± 7.6	28.1 ± 4.1	25.3 ± 4.3

*Note*: Data are presented as mean ± SD (*n* = 6).

Abbreviations: ALT, alanine transaminase; AST, aspartate transaminase; BUN, blood urea nitrogen; HCT, hematocrit; HGB, hemoglobin; MCH, mean corpuscular hemoglobin; MCHC, mean corpuscular hemoglobin concentration; MCV, mean corpuscular volume; PLT, blood platelet count; RBC, red blood cell count; SCr, serum creatinine; WBC, white blood cell count.

## 4 CONCLUSIONS

We developed DOX‐ and MTX‐adsorbed TPP‐decorated HSA NCs to enhance the therapeutic efficacy of the MAP regimen for osteosarcoma. DOX or MTX adsorption to HSA‐TPP induced self‐assembly of NC structures with high colloidal stability in serum, solubilizing the hydrophobic drugs up to therapeutically meaningful concentrations. The developed NCs exhibited a significantly augmented affinity to the bone TME, which was demonstrated using various in vitro and in vivo osteosarcoma models. The HSA‐TPP NC‐assisted MAP regimen consisting of a combination of DOX/HSA‐TPP with free CDDP and MTX/HSA‐TPP treatments displayed an enhanced synergistic effect in suppressing osteosarcoma cells, whereas conventional solution MAP showed antagonism in CI values. All these results translated into an improved antitumor efficacy in an orthotopic osteosarcoma‐xenografted mouse model; the mice in the HSA‐TPP MAP group exhibited a significant reduction in the osteosarcoma volume without showing any noticeable systemic toxicities when compared with those in the solution MAP and HSA MAP groups. Thus, the HSA‐TPP NCs‐based MAP regimen may be a promising strategy for osteosarcoma treatment.

## AUTHOR CONTRIBUTIONS

**So‐Yeol Yoo:** Conceptualization (lead); data curation (lead); formal analysis (lead); methodology (equal); software (lead); visualization (equal); writing – original draft (lead). **Yong‐Hyeon Mun:** Conceptualization (lead); data curation (equal); methodology (lead); software (equal); writing – original draft (lead). **Nae‐Won Kang:** Data curation (lead); formal analysis (lead); validation (lead); visualization (lead); writing – original draft (equal). **Jang Mo Koo:** Methodology (supporting); software (supporting). **Dong Hwan Lee:** Methodology (supporting); validation (supporting). **Ji Hoon Yoo:** Methodology (supporting); visualization (supporting). **Sang Min Lee:** Investigation (supporting); visualization (supporting). **Seokjin Koh:** Software (supporting); validation (supporting). **Jong Chan Park:** Formal analysis (supporting); visualization (supporting). **Taejung Kim:** Formal analysis (supporting); methodology (supporting). **Eun Kyung Shin:** Methodology (supporting); software (supporting). **Han Sol Lee:** Investigation (supporting); methodology (supporting); visualization (supporting). **Jaehoon Sim:** Methodology (supporting); software (supporting). **Keon Wook Kang:** Data curation (supporting); formal analysis (supporting); validation (supporting); visualization (supporting). **Sang Kyum Kim:** Data curation (supporting); formal analysis (supporting); investigation (supporting); software (supporting); visualization (supporting). **Cheong‐Weon Cho:** Formal analysis (supporting); software (supporting); validation (supporting); visualization (supporting). **Myeong Gyu Kim:** Formal analysis (lead); investigation (equal); methodology (equal); writing – review and editing (equal). **Dae‐Duk Kim:** Data curation (equal); formal analysis (lead); funding acquisition (lead); resources (lead); supervision (lead); visualization (equal); writing – review and editing (equal). **Jae‐Young Lee:** Conceptualization (lead); data curation (equal); formal analysis (lead); funding acquisition (lead); methodology (equal); project administration (lead); resources (lead); supervision (lead); visualization (equal); writing – review and editing (lead).

## CONFLICT OF INTEREST

The authors declare no conflict of interest.

### PEER REVIEW

The peer review history for this article is available at https://publons.com/publon/10.1002/btm2.10472.

## Supporting information


**DATA S1.** Supporting Information

## Data Availability

The data that support the findings of this study are available from the corresponding author upon reasonable request.
