# Supplementary material for: Enhancement of the therapeutic efficacy of the MAP regimen using thiamine pyrophosphate‐decorated albumin nanoclusters in osteosarcoma treatment
Source: Bioeng Transl Med. 2022 Dec 26;8(6):e10472. doi: 10.1002/btm2.10472 (PMC10658614; doi:10.1002/btm2.10472)
Supplement: Supplementary file 1 — DATA S1. Supporting Information [file BTM2-8-e10472-s001.docx]

Supporting Information

**Enhancement of the Therapeutic Efficacy of the MAP Regimen using Thiamine Pyrophosphate-decorated Albumin Nanoclusters in Osteosarcoma Treatment**

*So-Yeol Yoo^1^, Yong-Hyeon Mun^1^, Nae-Won Kang^2^, Jang Mo Koo^1^, Dong Hwan Lee^1^,*

*Ji Hoon Yoo^1^,* *Sang Min Lee^1^, Seokjin Koh^1^, Jong Chan Park^1^, Taejung Kim^1^, Eun Kyung Shin^2^*,

*Han Sol Lee^1^, Jaehoon Sim^1^, Keon Wook Kang^2^, Sang Kyum Kim^1^, Cheong-Weon Cho^1^,*

*Myeong Gyu Kim^3^, Dae-Duk Kim^2^, and Jae-Young Lee^1^*

^1^College of Pharmacy, Chungnam National University, Daejeon 34134, Republic of Korea

^2^College of Pharmacy and Research Institute of Pharmaceutical Sciences, Seoul National University, Seoul 08826, Republic of Korea

^3^College of Pharmacy and Graduate School of Pharmaceutical Sciences, Ewha Womans University, Seoul 03760, Republic of Korea

**Correspondence**

Myeong Gyu Kim, College of Pharmacy and Graduate School of Pharmaceutical Sciences, Ewha Womans University, Seoul 03760, Republic of Korea

Tel.: +82 02 3277 3102

E-mail: kimmg@ewha.ac.kr

Dae-Duk Kim, College of Pharmacy and Research Institute of Pharmaceutical Sciences, Seoul National University, Seoul 08826, Republic of Korea

Tel.: +82 2 880 7870

E-mail: ddkim@snu.ac.kr

Jae-Young Lee, College of Pharmacy, Chungnam National University, Daejeon 34134, Republic of Korea.

Tel.: +82 42 821 5935

E-mail: jaeyoung@cnu.ac.kr

So-Yeol Yoo, Yong-Hyeon Mun, Nae-Won Kang contributed equally to this study

**Keywords**: osteosarcoma, thiamine pyrophosphate, albumin nanoclusters, hydroxyapatites, MAP regimen

**Supplementary Tables**

**Table S1**. Optimized LC-MS/MS parameters for the detection of DOX, MTX, and docetaxel (IS).

| Compound | Precursor ion (*m/z*) | Product  ion (*m/z*) | DP (V) | EP (V) | CEP (V) | CE (eV) | CXP (V) |
| --- | --- | --- | --- | --- | --- | --- | --- |
| DOX | 544.0 | 397.2 | 20 | 2 | 18 | 17 | 8 |
| MTX | 455.3 | 175.1 | 42 | 4 | 14 | 52 | 3 |
| Docetaxel | 830.2 | 549.4 | 85 | 9 | 36 | 23 | 8 |

Abbreviations: IS, internal standard; DP, declustering potential; EP, entrance potential; CEP, cell entrance potential; CE, collision energy; and CXP, collision cell exit potential.

**Table S2**. Concentration ratio of DOX/MTX/CDDP used in the *in vitro* combination therapy.

| **DOX (µg/mL)** | **MTX (µg/mL)** | **CDDP (µg/mL)** | **Total drug concentration (µg/mL)** |
| --- | --- | --- | --- |
| 5 | 10 | 3 | 18 |
| 1.25 | 2.5 | 0.75 | 4.5 |
| 0.5 | 1 | 0.3 | 1.8 |
| 0.125 | 0.25 | 0.075 | 0.45 |
| 0.05 | 0.1 | 0.03 | 0.18 |
| 0.0125 | 0.025 | 0.0075 | 0.045 |
| 0.005 | 0.01 | 0.003 | 0.018 |
| 0.00125 | 0.0025 | 0.00075 | 0.0045 |

**Table S3**. Model fitting results of release profiles.

| **Groups** | **pH** | **First-order with F_max_** | | | **Hopfenberg** | | | **Korsmeyer–Peppas** | | | **Peppas–Sahlin** | | | |
| --- | --- | --- | --- | --- | --- | --- | --- | --- | --- | --- | --- | --- | --- | --- |
|  |  | **R^2^** | **k** | **F_max_** | **R^2^** | **k_HB_** | **n** | **R^2^** | **k_KP_** | **n** | **R^2^** | **k_1_** | **k_2_** | **m** |
| DOX/HSA | 6.7 | 0.9721 | 0.0467 | 56.30 | 0.8143 | 0.00001 | 2412 | 0.9737 | 7.967 | 0.4461 | 0.9905 | 5.789 | –0.1432 | 0.6354 |
|  | 7.4 | 0.9585 | 0.0531 | 37.97 | 0.6652 | 0.00001 | 774.1 | 0.9801 | 6.246 | 0.4161 | 0.9939 | 4.900 | –0.1456 | 0.5796 |
| DOX/HSA-TPP | 6.7 | 0.9622 | 0.0486 | 54.79 | 0.7775 | 0.00001 | 1623 | 0.9855 | 8.421 | 0.4268 | 0.9965 | 6.607 | –0.1856 | 0.5857 |
|  | 7.4 | 0.9263 | 0.0540 | 40.51 | 0.6660 | 0.00001 | 1322 | 0.9925 | 6.703 | 0.4137 | 0.9956 | 6.110 | –0.1611 | 0.4989 |
| MTX/HSA | 6.7 | 0.9979 | 0.2331 | 84.00 | 0.8114 | 0.0001 | 2302 | 0.8055 | 34.32 | 0.2243 | 0.9321 | 29.38 | –2.395 | 0.4522 |
|  | 7.4 | 0.9979 | 0.2227 | 85.24 | 0.8491 | 0.0001 | 1836 | 0.8111 | 33.84 | 0.2309 | 0.9374 | 28.56 | –2.227 | 0.4623 |
| MTX/HSA-TPP | 6.7 | 0.9988 | 0.2308 | 95.09 | 0.9846 | 0.0001 | 2009 | 0.8127 | 38.78 | 0.2246 | 0.9395 | 33.12 | –2.686 | 0.4530 |
|  | 7.4 | 0.9986 | 0.2311 | 93.48 | 0.9729 | 0.0001 | 1623 | 0.8015 | 38.14 | 0.2243 | 0.9335 | 32.41 | –2.616 | 0.4560 |

The coefficient of determination is presented as R^2^. F_max_ is the maximum cumulative release, and k, k_HB_, k_KP_, k_1_, and k_2_ are the release rate constants of the models.

**Table S4**. IC_50_ and predicted CI at 50% drug effect level of the solution- and NC-based MAP regimen.

| **Treatment (incubation time)** | **IC_50_ (µg/mL)** | **CI_50_** |
| --- | --- | --- |
| DOX solution (48 h) + CDDP solution (48 h) + MTX solution (24 h) | 2.158 ± 0.523 | 1.570 |
| DOX/HSA NCs (48 h) + CDDP solution (48 h) + MTX/HSA NCs (24 h) | 0.371 ± 0.315 | 0.358 |
| DOX/HSA-TPP NCs (48 h) + CDDP solution (48 h) + MTX/HSA-TPP NCs (24 h) | 0.807 ± 0.541 | 0.806 |

**Table S5.** Pharmacokinetic paramters of DOX- or MTX-loaded NCs (cf. Figure S4).

| **Parameters** | **DOX/HSA** | **DOX/HSA-TPP** | **MTX/HSA** | **MTX/HSA-TPP** |
| --- | --- | --- | --- | --- |
| T_1/2_ (h) | 2.15 ± 0.6 | 1.94 ± 0.31 | 1.31 ± 0.73 | 0.84 ± 0.24 |
| C_max_ (ng/mL) | 1021 ± 196 | 363 ± 53 | 1988 ± 287 | 1751 ± 273 |
| AUC_last_ (hr∙ng/mL) | 2122 ± 489 | 827 ± 122 | 3317 ± 583 | 2856 ± 546 |
| V_d_ (mL/kg) | 7169 ± 2077 | 16472 ± 4631 | 5933 ± 4149 | 4139 ± 542 |
| CL (mL/hr/kg) | 2340 ± 507 | 5824 ± 834 | 3013 ± 493 | 3528 ± 591 |

**Supplementary Figures**


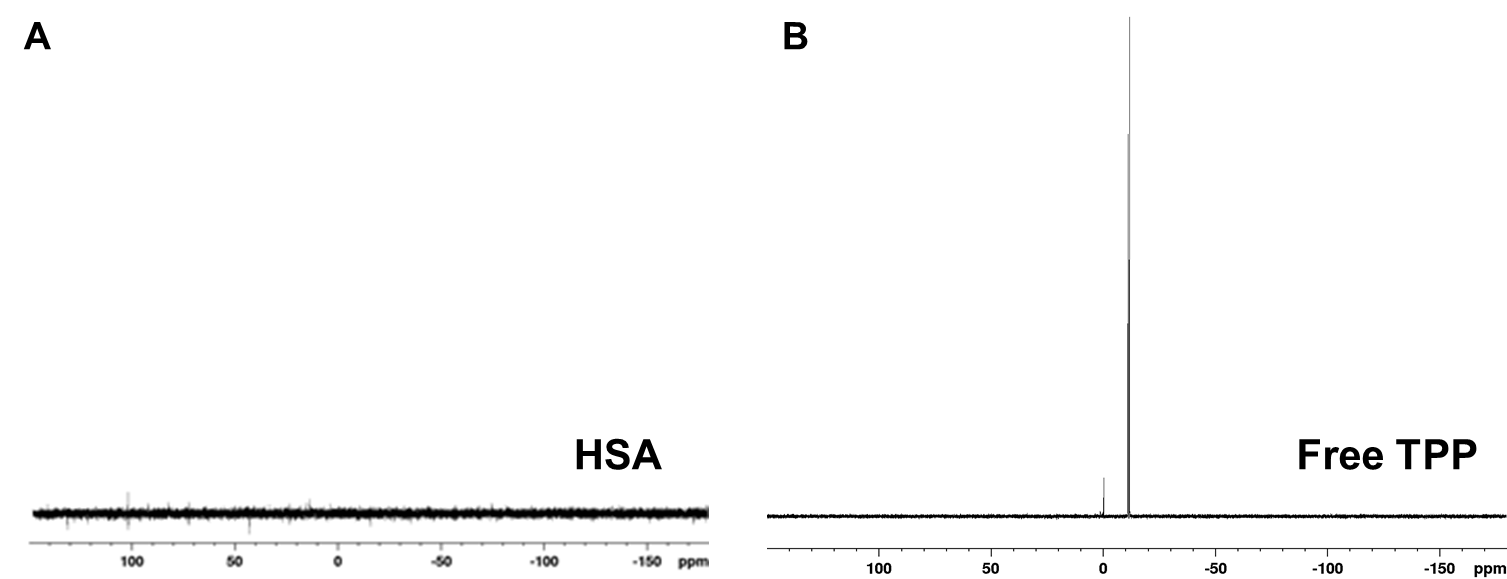


**Figure S1**. ^31^P-NMR spectra of (A) native HSA and (B) free TPP. No significant peaks were detected in the HSA spectrum.


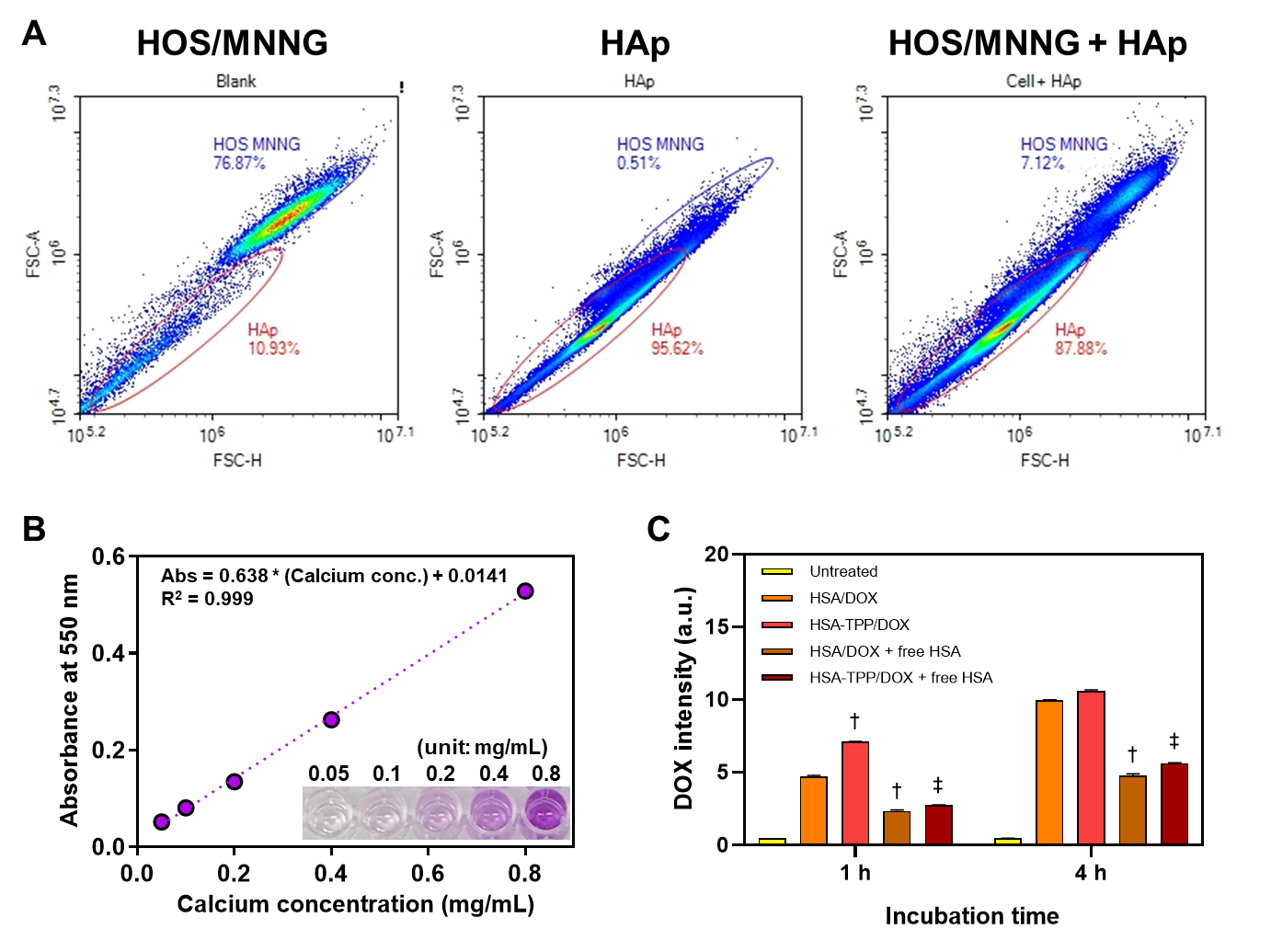


**Figure S2**. (A) Adsorption of HAp to HOS/MNNG cells was investigated using flow cytometry. Singlet gates of HOS/MNNG and HAp were set based on FSC-A vs. FSC-H distribution. The population of cell debris correponding to the low-FSC subset included in the HAp gate was negligible in the HOS/MNNG + HAp sample (~1.01%). (B) The amount of Ca-ARS complex was quantified based on spectrophotometry, where a calibration curve was constructed using Ca(H_2_PO_4_)_2_ standard samples. (C) Cellular uptake study performed in conventional monolayer-cultured HOS/MNNG cells using flow cytometry. The DOX fluorescence intensity was measured after a 1- or 4-h incubation with the NCs at a DOX concentration of 5 μg/mL. Free HSA was used as a competitive inhibitor against albumin receptors. ^†^*p* < 0.001 compared to HSA/DOX; ^‡^*p* < 0.0001 compared to HSA-TPP/DOX.

**Figure S3**. Cytotoxicity of free CDDP against HOS/MNNG cells. The cell viability was measured using Cell Counting Kit-8.


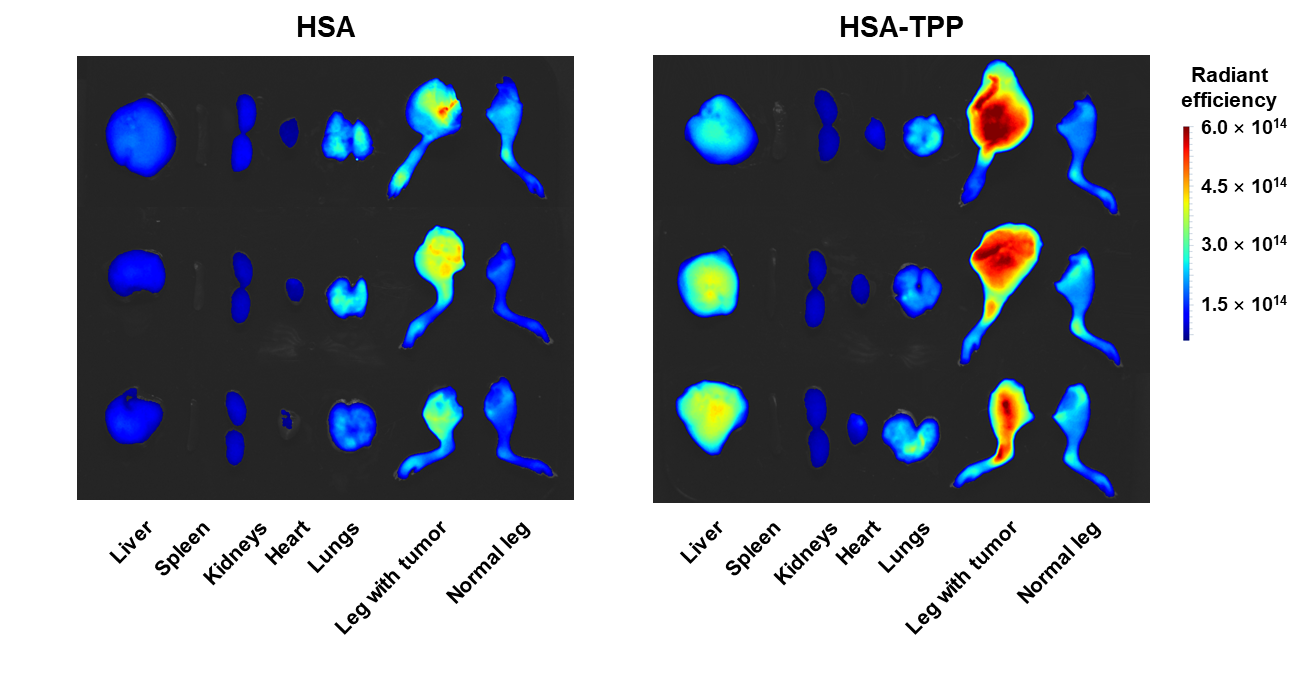


**Figure S4.** *Ex vivo* NIRF images of major organs and legs at 24 h post-injection (*n* = 3). Images of all subjects are presented (cf. Figure 4D).


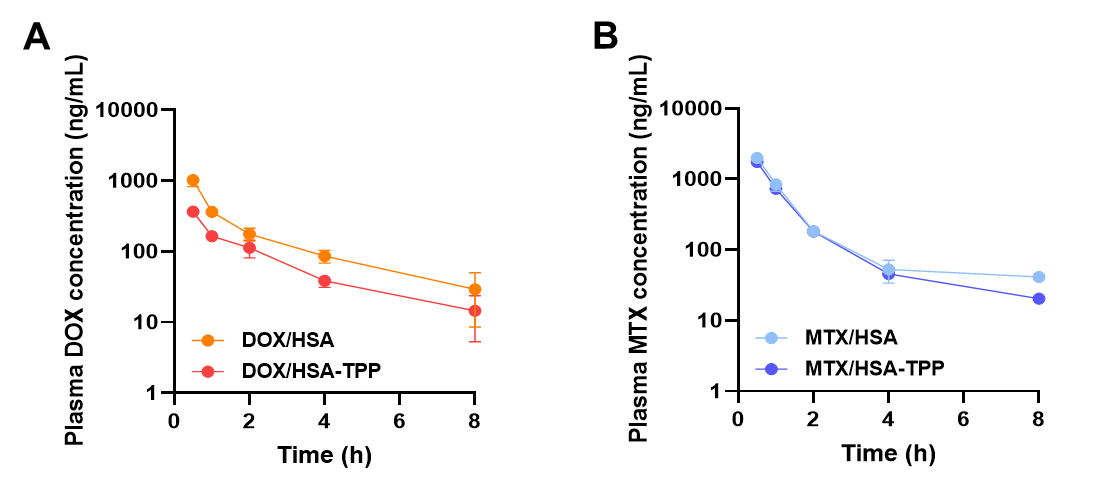


**Figure S5.** Plasma drug concentration vs. time profiles of the developed NCs in Balb/c mice (*n* = 6). Each formulation was injected intravenously at a dose of 5 mg/kg for DOX-loaded NCs or 10 mg/kg for MTX-loaded NCs. Corresponding pharmacokinetic parameters are presented in Table S5.


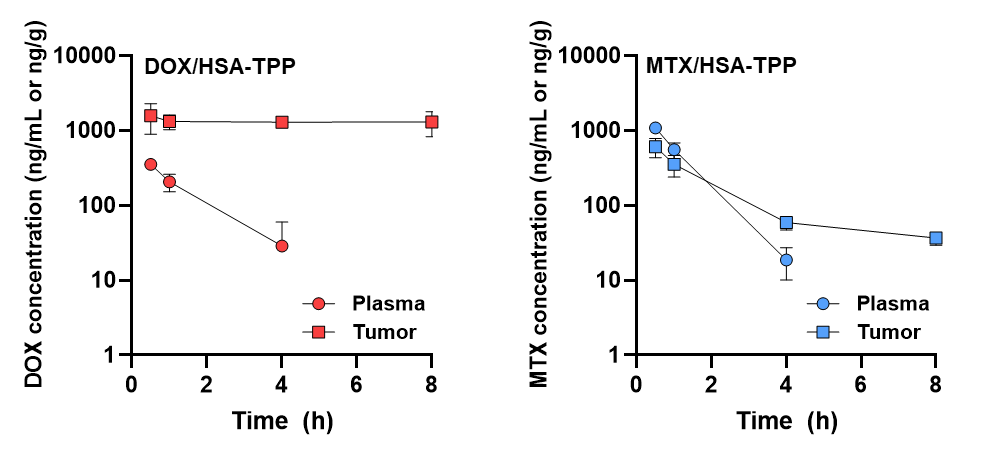


**Figure S6.** Drug concentration vs. time profiles of plasma and tumor tissues after an intravenous administration of DOX/HSA-TPP (5 mg/kg) or MTX/HSA-TPP (10 mg/kg) in the orthotopic osteosarcoma xenograft mice (*n* = 4).

**Figure S7**. Relative expression of anti-apoptotic proteins in the tumor after three cycles of HSA-TPP NC-assisted MAP as compared to that in the untreated tumors. The values next to the scale bar indicate fold-changes.


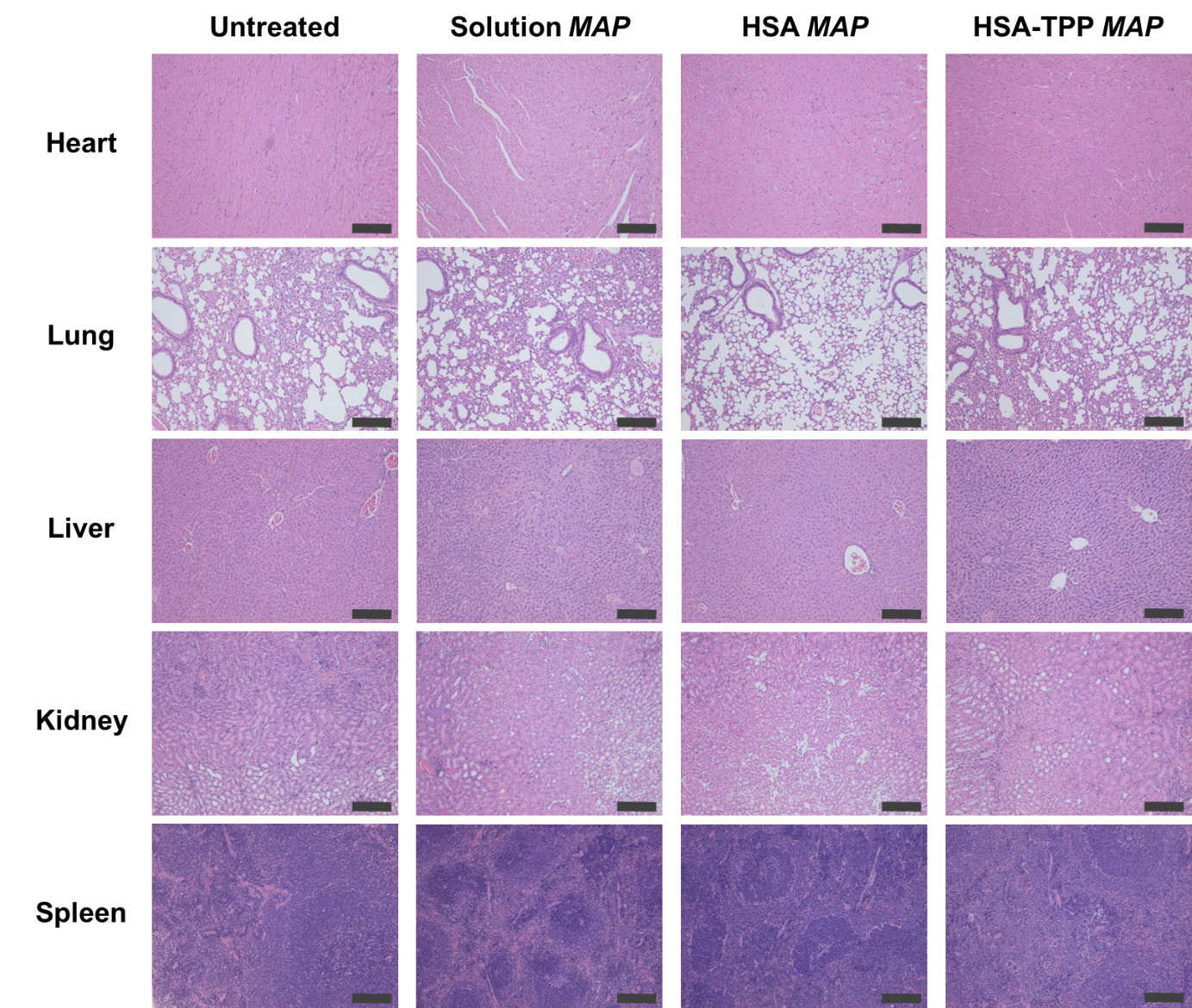


**Figure S8**. Evaluation of off-target toxicities after three cycles of MAP. Histological analyses were performed on the major organs after H&E staining. The length of the scale bars is 100 μm.
